# The impact of chemotherapy on floating vs. attached colorectal cancer cells: An *in-vitro* study

**DOI:** 10.3389/fonc.2026.1697776

**Published:** 2026-02-04

**Authors:** Veria Khosrawipour, Agata Mikołajczyk-Martinez, Carolina Khosrawipour, Patrycja Salata, Zdzisław Kiełbowicz, Ewa Nicpon, Przemyslaw Przadka, Zhiwen Zhang, Wojciech Kielan, Shiri Li, Jacek Bania, Kamila Kuzniak, Kacper Zieliński, Mouhamed El-Aita, Wolfram T. Knoefel

**Affiliations:** 1 Peritoneal Cancer Research Group Wroclaw (PCRGW), Wroclaw University of Environmental and Life Sciences, Wroclaw, Poland; 2 University Centre of General and Oncological Surgery, Wroclaw Medical University, Wroclaw, Poland; 3 Department of Biochemistry and Molecular Biology, Wroclaw University of Environmental and Life Sciences, Wroclaw, Poland; 4 Department of Surgery (A), Heinrich-Heine-University, Medical Faculty and University Hospital Duesseldorf, Universitatsklinikum Dusseldorf, Düsseldorf, Germany; 5 Department of Food Hygiene and Consumer Health Protection, Wroclaw University of Environmental and Life Sciences, Wroclaw, Poland; 6 Department and Clinic of Surgery, Faculty of Veterinary Medicine, Wroclaw University of Environmental and Life Sciences, Wroclaw, Poland; 7 School of Pharmacy, Key Laboratory of Small Drug Delivery (Ministry of Education) & National Key Laboratory of Complex Drug Formulations for Overcoming Delivery Barriers, Fudan University, Shanghai, China; 8 Division of Colorectal Surgery, Department of Surgery, Cedars-Sinai, Los Angeles, CA, United States; 9 School of Veterinary Medicine, Wroclaw University of Environmental and Life Sciences, Wroclaw, Poland; 10 Department of Plastic Surgery, Evangelisches Stift Sankt Martin, Koblenz, Germany

**Keywords:** adhesion, chemotherapy, colorectal cancer, HT-29 cells, peritoneal metastasis

## Abstract

**Background:**

Peritoneal metastasis (PM) is a highly aggressive, hard-to-treat malignant disease which has continuously increased in incidence and clinical urgency. The progression of peritoneal metastasis (PM) is primarily driven by the spread of intraperitoneal cancer cells through initial surface adhesion, followed by growth and local invasion. Therefore, prevention of this initial adhesion may help to inhibit metastatic formation. By using a novel *in-vitro* PM model, this study investigates the inhibitory effect of chemotherapy on floating colorectal cancer cells compared to attached cells. The study intends to evaluate whether floating cancer cells are more sensitive to chemotherapy than attached ones.

**Methods:**

HT-29 colorectal cancer cells were exposed to oxaliplatin (OX) and doxorubicin *in-vitro* while in an attached vs. floating state. Cell viability was assessed using MTS assay, and intracellular chemotherapy uptake was measured via flow cytometry. Both groups were compared regarding metastatic colony formation using a novel *in-vitro* peritoneal metastasis model created by laparoscopically harvesting peritoneal progenitor cells.

**Results:**

OX exposure to floating cancer cells significantly reduced cell adhesion and viability compared to OX exposure to attached cells (p < 0.05). Flow cytometry revealed that this effect was not due to increased chemotherapy uptake in floating cells. In the *in-vitro* peritoneal cancer cell model, floating cancer cells treated with OX showed a markedly reduced ability to form cancer metastasis.

**Conclusions:**

Chemotherapy administered to cancer cells prior to full surface adhesion strongly impairs cancer cell adhesion, growth, and expansion. This phenomenon may partly explain the favorable clinical outcomes observed with (neo)adjuvant chemotherapy before and after complete cancer resection in PM and other cancer types. Floating cells seem to be more sensitive to chemotherapeutic agents than attached cells. Delaying and targeting specifically the ability of cancer cells to attach could help enormously in the treatment of many cancer manifestations such as PM. Further research is needed to confirm these findings in other cancer entities and to explore their potential clinical applications.

## 1 Introduction

Cancer metastases generally follow a characteristic pattern in which cells detach from one or more primary tumor nodules and disseminate into the bloodstream, lymphatic system, or a body cavity, where they may remain free-floating before adhering to a new surface (1–3). Such target surfaces may include endothelial, urothelial, peritoneal, or pleural linings. Successful adhesion is a prerequisite for subsequent proliferation, local invasion, and potential destruction of the surrounding tissue (4). While the underlying mechanisms remain poorly understood, increasing evidence indicates that cell adhesion plays a crucial role in modulating cancer cell metabolism (5, 6). Non-adherent, freely floating cancer cells generally fail to proliferate unless they are organized into larger cellular aggregates. In the absence of such clustering, these cells may persist in a dormant state or undergo apoptosis. A lack of adhesion to specific extracellular matrix (ECM) components has shown to trigger apoptotic pathways (7).

On the other hand, adherent cancer cells typically exhibit enhanced stability, resistance to stress, and increased proliferation rates (8, 9). It remains unclear whether this requirement for attachment to either a multicellular aggregate or a substrate is partially attributable to their developmental programming and, consequently, to their epidermal or endodermal origin. Current data suggests that disruption of adhesion processes can promote apoptosis in cancer cells, thereby inhibiting the formation of metastatic nodules. This phenomenon may be of great significance for optimizing the timing and administration of therapeutic agents aimed at preventing and suppressing sustained metastatic progression (10, 11). Cell adhesion is a prerequisite for continued tumor growth; a finding consistently observed in *in-vitro* experiments. However, the impact of chemotherapeutic exposure on non-adherent, freely circulating tumor cells—and the implications of such exposure for their subsequent developmental trajectory — remains poorly understood. While this fundamental question has yet to be resolved, it may prove to be of substantial importance in the therapeutic management of peritoneal metastasis (PM).

In PM, surgical strategies aim to suppress, minimize, or potentially halt metastatic dissemination. Examples of these procedures include cytoreductive surgery (CRS) combined with hyperthermic intraperitoneal chemotherapy (HIPEC) as well as pressurized intraperitoneal aerosol chemotherapy (PIPAC) (12, 13). It is conceivable that freely circulating tumor cells exhibit limited chemotherapeutic uptake due to their potentially reduced metabolic activity. Conversely, their non-adherent state might increase membrane permeability, thereby enhancing drug uptake and potentially improving chemotherapeutic efficacy at this stage. Alternatively, the timing of chemotherapeutic exposure—whether during the floating or adherent phase—may exert no measurable influence on subsequent tumor progression. To address these fundamental questions, the present study was designed to evaluate the effects of chemotherapeutic exposure on non-adherent cancer cells and its consequences for subsequent tumor cell proliferation. Data obtained from floating cells were compared to those from adherent cells. Furthermore, to assess intracellular drug uptake, both groups underwent flow cytometric analysis, and the impact on metastatic nodule formation was investigated using a novel *in-vitro* peritoneal model.

## 2 Materials and methods

### 2.1 Experimental parts

- In the initial phase of the experiment, the proliferative capacity of colorectal cancer cells (HT-29) was assessed following oxaliplatin exposure during either the floating or the attached state. This procedure was subsequently repeated with the addition of EDTA as a supplementary agent. HT-29 cells were exposed to oxaliplatin (OX, Cat#O9512, Sigma-Aldrich, Merck KGaA, Darmstadt, Germany) just before transfer to a 24-well plate (2 groups) or they were subjected to exposure after transfer and adhesion (1 group). Another group was not treated with OX and was used as a control. Cell-viability was measured via 3-(4,5-dimethylthiazol-2-yl)-5-(3-carboxymethoxyphenyl)-2-(4-sulfophenyl)-2H-tetrazolium (MTS) essay.
- After repeating the experimental procedure (Oxd) as previously described, a portion of the culture medium was removed from the 24-well plate and transferred for further analysis using a fluorescence cell counter (CellDrop, DeNovix, Wilmington, DE, USA). Medium containing residual floating cells was subjected to cell counting and subsequently transferred to a separate well plate for further cultivation. Transfers were performed at 12, 16, and 24 hours after initial placement in the 24-well plate. Cells were then cultured for seven days with regular medium changes.
- In the third phase of the experiment, HT-29 cells were exposed to OX while in a detached state and subsequently transferred to wells containing a developing peritoneal surface layer, hereafter referred to as the *in-vitro* peritoneal model (IPM). Using the IPM, nodule formation was compared between unexposed HT-29 cells and HT-29 cells exposed to OX. Cultures were monitored by light microscopy during the follow-up period. OX was used as it is a commonly used chemotherapeutic agent for colorectal cancer.
- In the fourth phase of the experiment, HT-29 cells from part (A) were exposed to doxorubicin (Cat#D1515 Sigma-Aldrich), selected for its intrinsic fluorescence properties, either during the floating or the attached state. Cells were subsequently analyzed by flow cytometry to quantify intracellular doxorubicin uptake. Doxorubicin was used for flowcytometry as it is the only commonly used chemotherapeutic agent with fluorescence characteristics.

### 2.2 HT-29 colorectal cancer line

The human colorectal cancer cell line HT-29 (ATCC^®^ HTB-38™) was obtained from the Institute of Immunology and Experimental therapy (Wrocław, Poland). Cells were grown in Dulbecco’s modified Eagle’s medium (DMEM - high glucose, Cat#D6429, Sigma-Aldrich), supplemented with 10% heat-inactivated fetal bovine serum (FBS, Gibco, Cat# A5256701,Thermo Fisher Scientific, Poland), 2 mmol/L glutamine (Cat#8540), 100 IU/mL penicillin (Cat#P3032), and 100 μg/mL streptomycin (Cat#S6501, all purchased from Sigma-Aldrich; Merck KGaA) at 36°C in a humidified 5% CO_2_/air atmosphere.

Drug dosage: In this study, we used 15 Millimolar (mM) Ethylenediaminetetraacetic acid (EDTA, Cat#108454, Stanlab, Lublin, Poland), which was dissolved in water and sterilized through a 0.2 µm filter and 184mg/150 mL of OX (Oxaliplatin Medoxa, medac GmbH,Wedel, Germany) diluted in a 5% glucose solution. Exposure concentration was based on commonly used treatment dosage for HIPEC which corresponds a exposure concentration of (0.24mg/ml).

### 2.3 Cell culture

#### 2.3.1 Part A

Cells were seeded in 6-well plates (TC Plate 6 Well, Standard, F, Sarstedt AG & Co. KG, Germany) at a density of 5.6 × 10^5^ cells per well and incubated under identical conditions. After three days of incubation, cells were trypsinized and detached from the wells by gentle shaking. HT-29 cells were allocated into four groups: three experimental groups and one untreated control. All groups underwent the same trypsinization protocol, with trypsin neutralized by the addition of fresh culture medium. Two experimental groups (OXd and OXdA) were exposed to OX(exposure concentration 0.24mg/ml) for one hour prior to transfer into 24-well plates. In the OXd group, exposure occurred while cells were in suspension in an Eppendorf tube, whereas in the OXdA group, exposure was performed immediately prior to trypsinization while cells were still adherent.

All groups were centrifuged, and the supernatant containing dissolved OX was discarded. Fresh culture medium was added to the resulting cell pellets, which were subsequently transferred into 24-well plates. The third experimental group (OXa) was exposed to OX after seeding and confirmed attachment to the culture surface, as verified by visual inspection after 24 hours. OX exposure was performed for one hour, after which the medium was replaced in all groups. Cells were then cultured for an additional five days, with medium changes every two days. The entire experiment was repeated on three separate occasions to ensure reproducibility.

#### 2.3.2 Part B

The experimental setting was the same as in A1. HT-29 cells were transferred to 24-well plates as previously described and allowed to sediment. At 12, 16, and 24 hours, 50 µl of medium containing non-adherent, floating cells was removed for cell counting and characterization using a fluorescence cell counter. The remaining medium, containing the adherent fraction, was transferred to a separate 24-well plate for isolated growth assessment. After 10 days, cell viability in these wells was evaluated using the MTS assay.

#### 2.3.3 Part C

The study included three 65-day-old swine that had previously participated in an unrelated investigation. During that earlier study, diagnostic laparoscopy was performed, and residual peritoneal fluid was aspirated from the abdominal cavity. For the present work, 5 mL of peritoneal fluid from each swine was utilized. Peritoneal fluid collection was performed using a standard laparoscopic approach. The collected fluid was passed through a coarse filter to remove larger tissue fragments, followed by centrifugation at 1500 rpm for 5 min. The resulting cell pellets were resuspended in culture medium and transferred to 6-well plates. Cultures were maintained with medium changes every two days for eight days to establish the IPM model, with cellular growth monitored via light microscopy. On day 8, 10.000 HT-29 colorectal cancer cells were added to each well. Then, HT-29 cells were either exposed to OX for one hour prior to transfer or left untreated. Next, cultures were placed in the incubator and daily monitored. Half of the wells with the unexposed HT-29 cells were subjected to OX exposure after having established nodule formation.

#### 2.3.4 Part D

To evaluate potential differences in doxorubicin uptake between adherent and non-adherent HT-29 cells following trypsinization, cells were exposed to doxorubicin under two distinct experimental conditions. HT-29 cells were seeded 72 hours prior to the experiment at a density of 2 × 10^5^ cells per well. In the first condition, cells were detached using trypsin–EDTA (Cat#T4049, Sigma-Aldrich; Merck KGaA), and trypsin activity was neutralized with culture medium. Detached cells were washed with phosphate-buffered saline (PBS) and resuspended in 1 mL of high-glucose DMEM containing doxorubicin at a concentration of 2.5 µM. Cells were incubated for 1h at 37 °C in a humidified 5% CO_2_ atmosphere. Following incubation, cells were washed twice with PBS and resuspended in 0.3 mL of PBS.

In the second condition, the order was reversed, and adherent cells were first exposed to doxorubicin under identical medium and incubation conditions, washed twice with PBS, and subsequently trypsinized as described above before resuspension in 0.3 mL of PBS. An untreated control group was included. Flow cytometry analysis (BD FACS Lyric; Becton Dickinson, Franklin Lakes, NJ, USA) was used to quantify intracellular doxorubicin levels in the samples. A total of 10.000 events per sample, gated on forward scatter (FSC)-A versus side scatter (SSC)-A dot plots, were recorded. Cells were excited with a 640nm red laser, and fluorescence emission was detected using the R-phycoerythrin (PE) channel. Positive signal thresholds were determined relative to the autofluorescence of the negative controls. All experiments were independently performed three times.

### 2.4 MTS test

Cell proliferation assay (CellTiter 96^®^AQ_ueous_ One Solution assay, Promega, Poland) was performed according to the manufacturer’s instruction with modifications. Briefly, the medium was removed from each well and replaced by 0.3 mL of fresh DMEM. Next, an MTS-based reagent was added to each well and absorbance was measured at 490nm after 1hour of incubation at 36°C at 5% CO_2_ using a microplate reader (Tecan, Basel, Switzerland).

### 2.5 Microscopic analyses

The IPM was visually assessed using an inverted transmitted-light microscope (Primovert, Zeiss, Göttingen, Germany), with the aid of the FX-5000 version 1.0 program, installed on a connected computer.

### 2.6 Statistical analysis

All experiments were independently performed three times. Statistical analyses were conducted with Sigma Plot 12 (Systat Software Inc., California, USA). Probability *(p)* values were considered as follows: *=*p* < 0.05; **=*p* < 0.01; #=*p*>0.05, with*p*-value <0.05 considered to be statistically significant.

## 3 Results

### 3.1 Cell culture data

**Figure 1:**
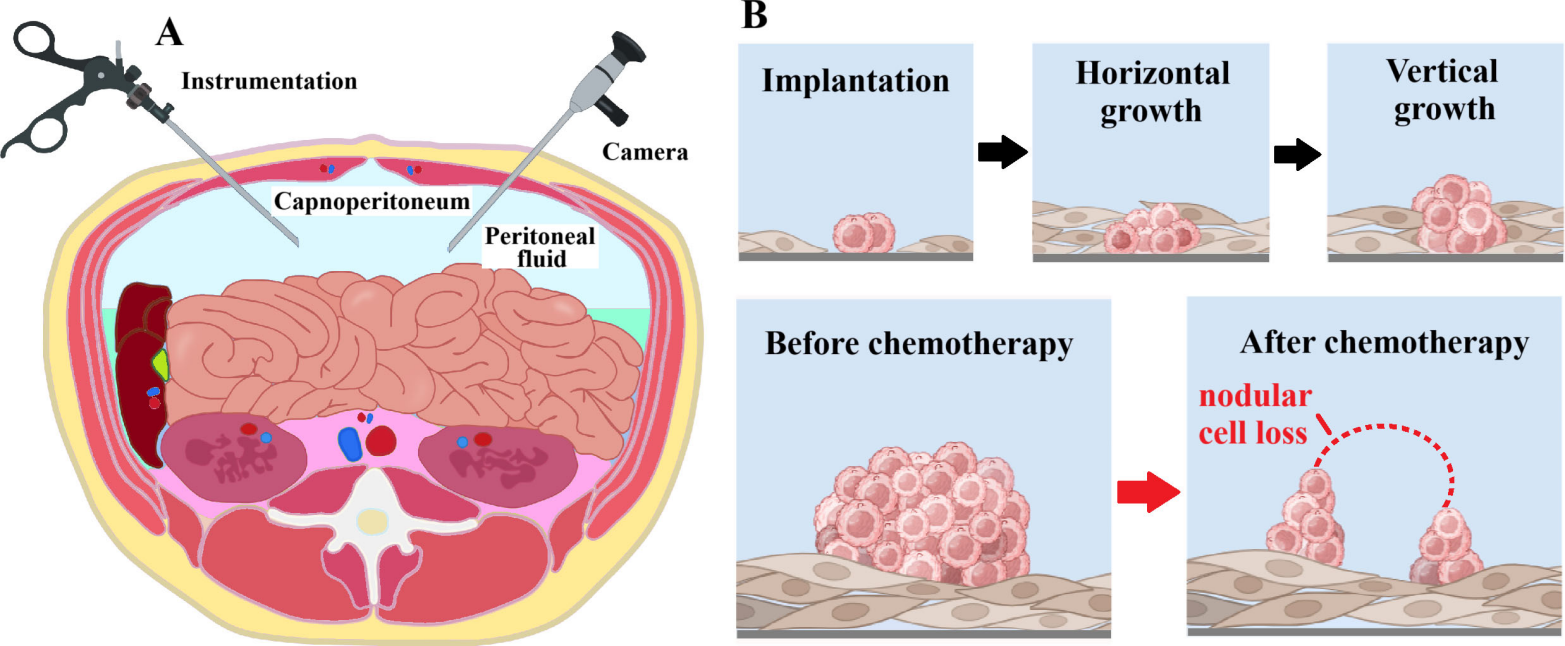
**(A)** Laparoscopic model of peritoneal fluid retrieval for sampling progenitor cells. **(B)** Sagittal section model demonstrating growth and changes of *in-vitro* PM following chemotherapy exposure.

**Figure 2:**
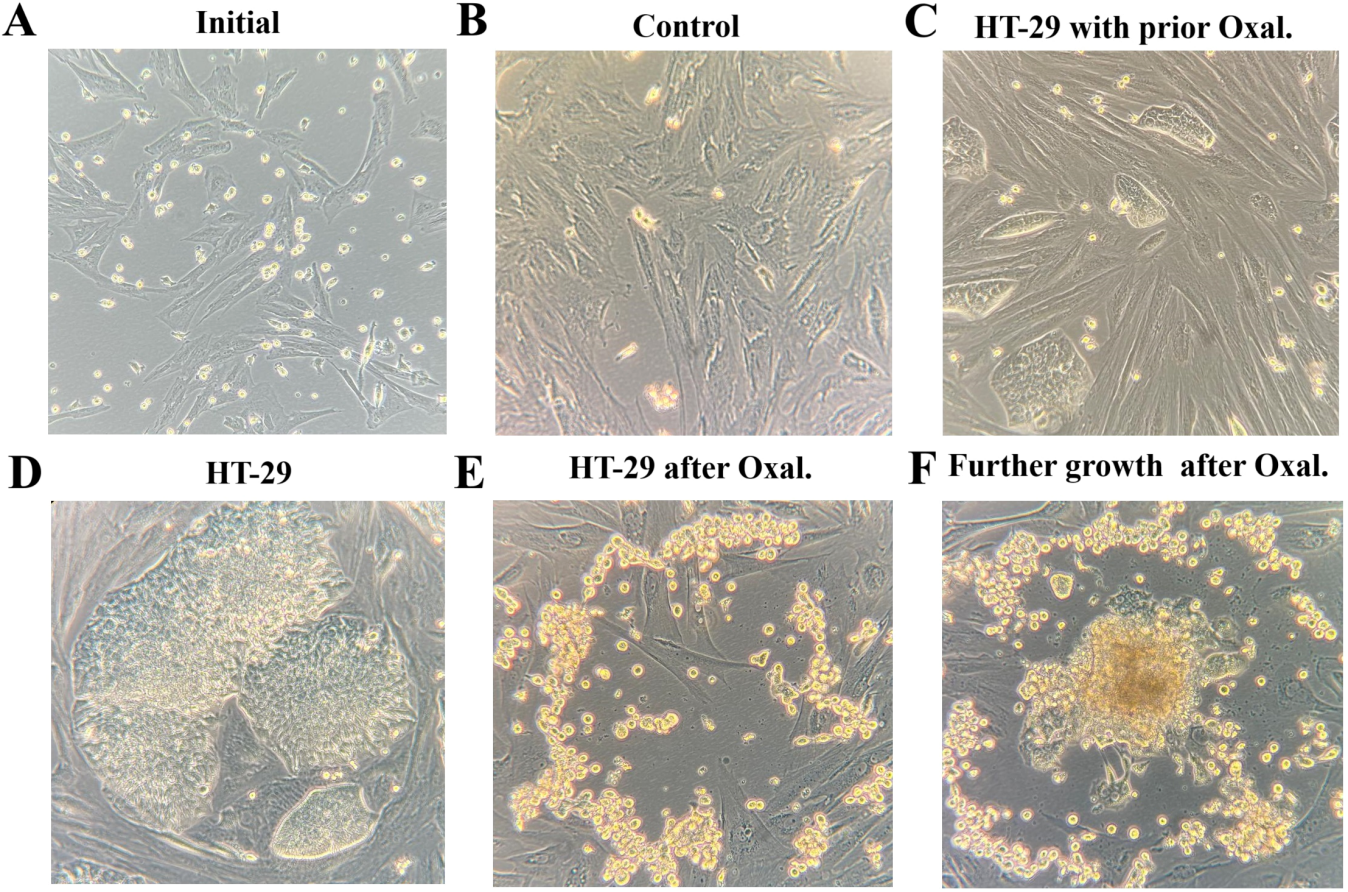
Inhibition of PM formation in the *in-vitro* peritoneal metastasis model (IPM). **(A)** Initial stage of peritoneal layer formation. **(B)** Advanced stage of peritoneal layer formation without cancer (Control). **(C)** Advanced stage of PM formation after prior exposure to oxaliplatin before transfer to IPM. **(D)** Advanced stage of PM formation with HT-29 cells. **(E)** Exposure to oxaliplatin following advanced stage of PM formation with HT-29 cells. **(F)** Regrowth after exposure to Oxaliplatin following advanced stage of PM formation with HT-29 cells with horizontal nodule formation.

**Figure 3:**
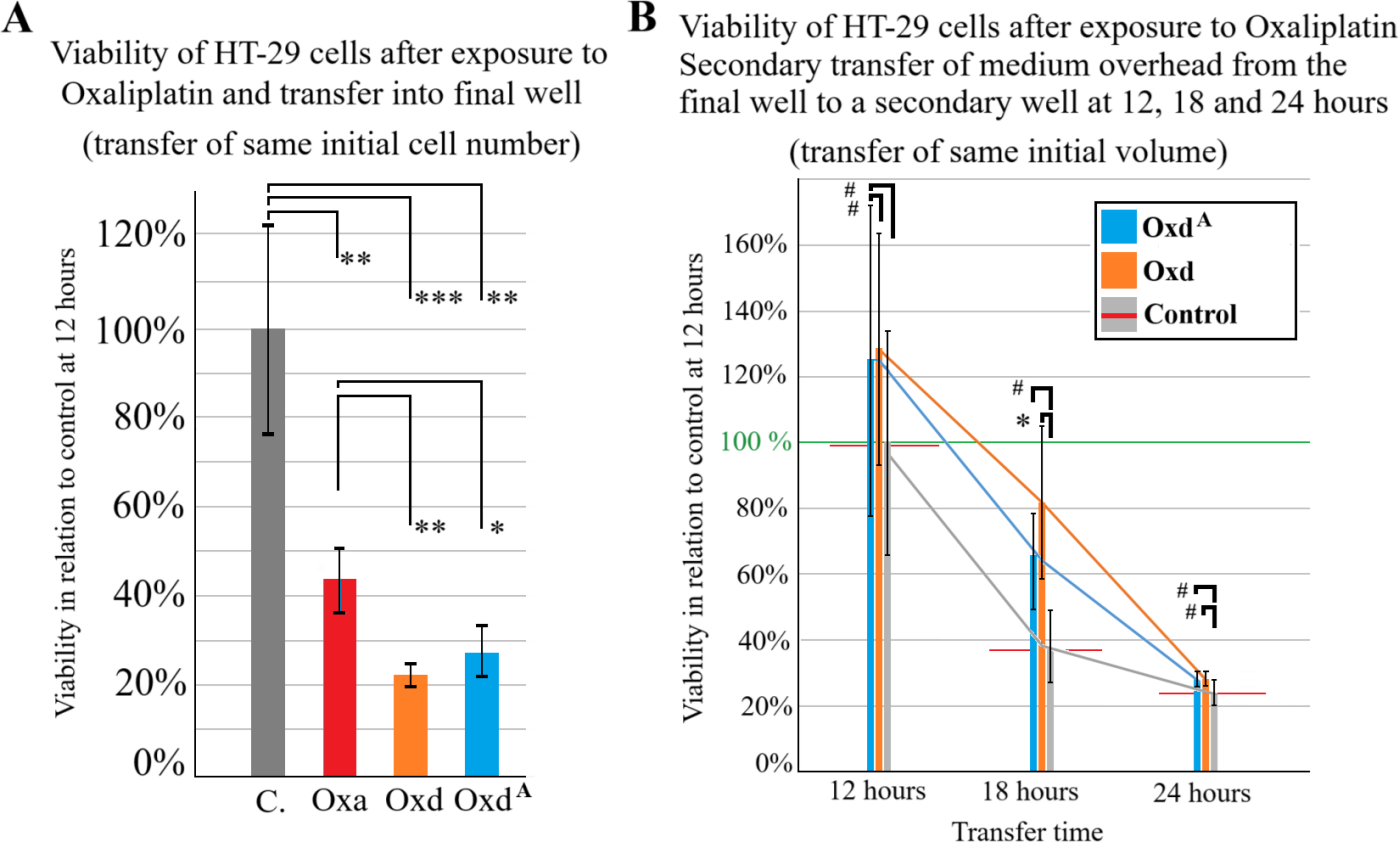
**(A)** Viability of HT-29 cells after previous exposure to Oxaliplatin. Cells were either exposed just before transfer (Oxd – Orange column and Oxd^A^-blue column) or while attached to the surface (Oxa-red column). Untreated cells were used as control (C.-grey column). All groups started with the same cell amount. **(B)** Viability of isolated HT-29 cells that were still afloat at 12-, 18- and 24-hours following transfer into the well. Viability of inhibited cells was compared to the control group unexposed to chemotherapy. P values are indicated with *P < 0.05, **P < 0.01, ***P < 0.001, and ^#^P >0.05.

The experimental workflow included harvesting progenitor cells, transferring them into the *in-vitro* PM model, and monitoring their growth (**Figures 1**, **2**). All procedures were successfully performed. In part A of the experiments, results demonstrated that the timing of OX exposure — whether HT-29 cells were adherent or non-adherent — significantly affected cell viability. Viability of HT-29 cells exposed to OX while adherent to the culture surface was markedly reduced compared with untreated controls (*p* < 0.01; **Figure 3A**). Notably, viability was further decreased when these treated adherent cells were compared to cells exposed to OX prior to transfer (*p* < 0.05). Inhibition of subsequent adhesion to the surface was not significantly different between cells exposed in the floating versus adherent state (OXd vs. OXdA, *p* > 0.05; **Figure 3A**). Overall, OX treatment led to a general suppression of adhesion, with cells remaining in a floating state within culture wells.

In the second part of experiment A, HT-29 cells exposed to EDTA failed to adhere under any condition and displayed minimal growth. Due to the absence of measurable differences between EDTA-treated groups, these data were not included in the figures. In experiment B, some of the medium from the 24-well plates was transferred onto a separate well. This occurred at 12, 18 and 24 hours after prior transfer as some cells were still afloat. The transferred cells were left to grow in the new well.

**Figure 4:**
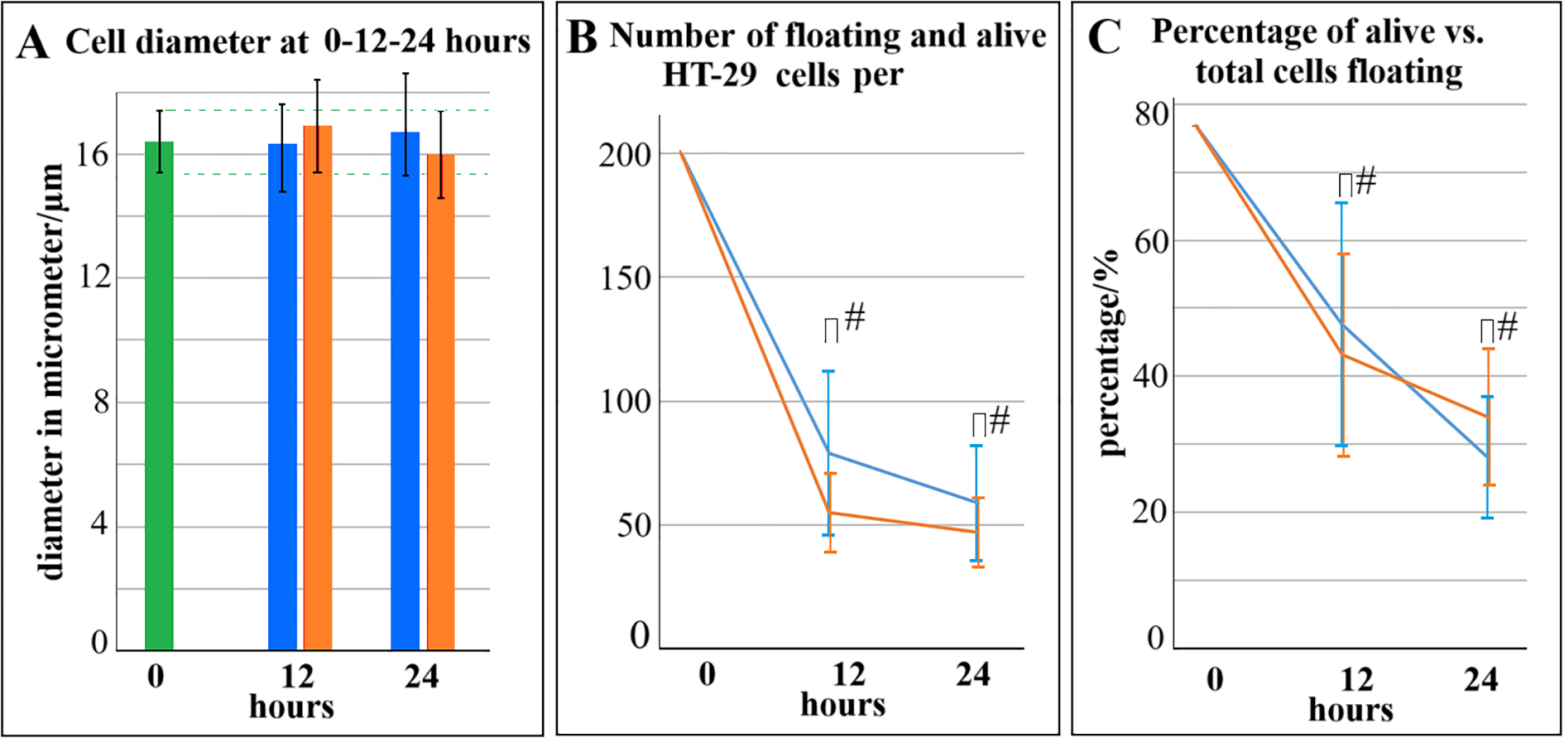
Characteristics of Oxd and Oxd^A^ cells in the medium after prior transfer into the well after 12 and 24 hours. **(A)** Cell diameter remains constant while absolute cell numbers per visual - counting field continually decrease for both groups **(B)** Same applies to the relative number of viable cells in relation to the total cell count. P values are indicated with ^#^P >0.05.

Viability numbers did not differ much for the groups (12h, 18h or 24 h) (**Figure 3B**). The only exception was Oxd vs. Control at 18 hours with a significant higher viability than control. It is noteworthy that the range of viability was quite fluctuating, resulting in a wide standard deviation for this experimental setting. In most cases, inhibited adhesion did not automatically result in HT-29 cells to be carried away and growth elsewhere in larger numbers than the untreated control. When comparing cell diameters, the number of viable floating cells and relative number of viable cells with each other in the Oxd^A^ and Oxd groups, no significant differences was noted (**Figures 4A–C**).

### 3.2 Peritoneal metastasis culture

In experiment C, the establishment of simulated peritoneal metastatic nodules in the IPM was visibly impaired when HT-29 cells were exposed to OX prior to transfer. In IPM without exposure to OX, cancer nodules just grew denser with time (**Figure 2A** versus **Figure 2B**). The growth in unexposed HT-29 cells was faster and nodules were much larger at corresponding times (**Figure 2C** vs. **Figure 2D**). At this point, it is important to mention that these nodules were not macroscopically detectable. When these previously unexposed HT-29 nodules were exposed to OX in the IPM model, nodules lost their compact structure and flattened out. Viable HT-29 cells remained on the margins of nodules (**Figure 2E**). When observing growth patterns after prior significant loss of HT-29 cells due to OX exposure, regrowth was observed predominantly vertically rather than horizontally as it did before exposure to oxaliplatin (**Figure 2D** versus **Figure 2F**).

### 3.3 Flow cytometry

**Figure 5:**
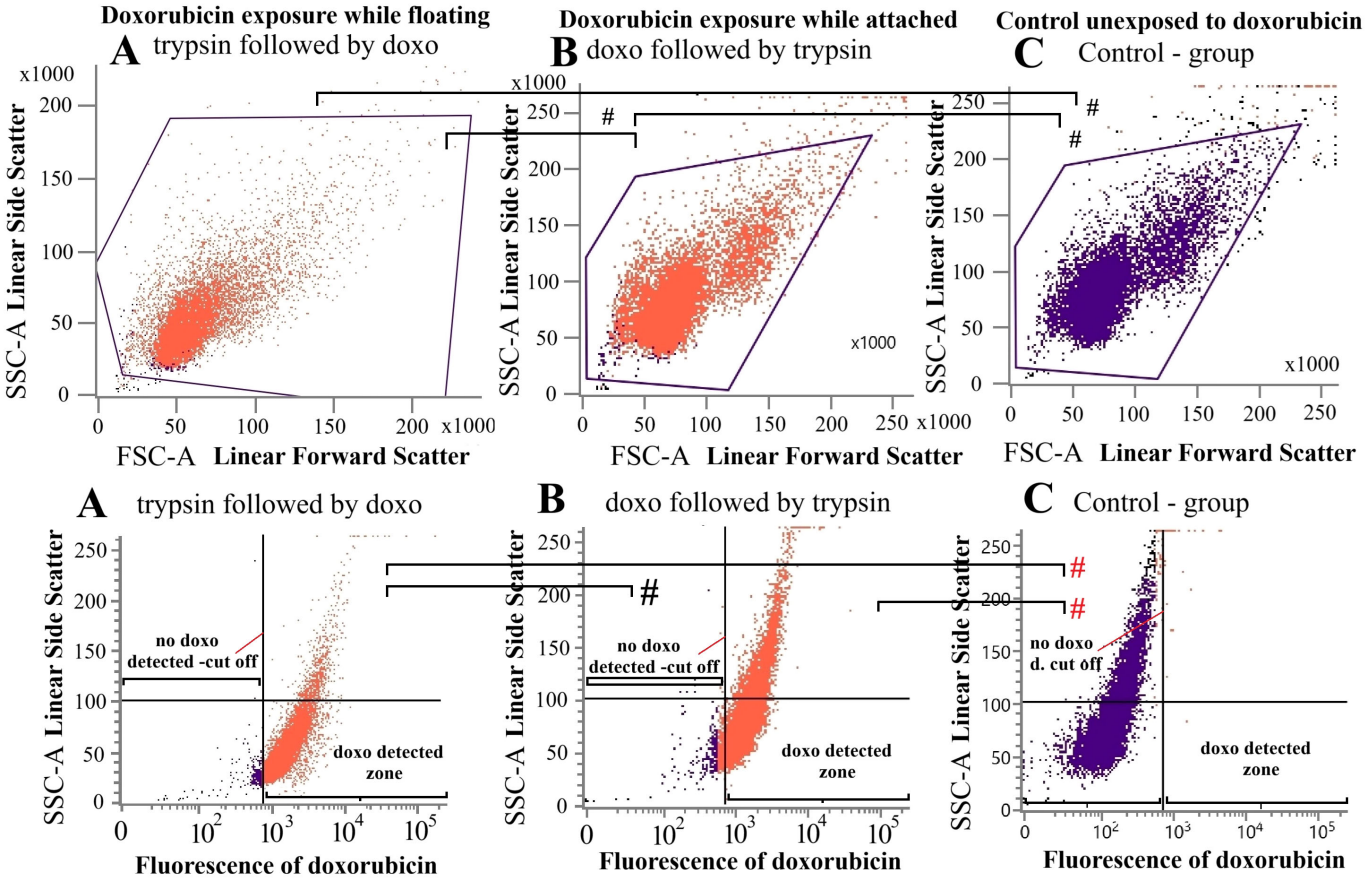
Flow-cytometry, cellular doxorubicin uptake (orange dots) of detached **(A)** and undetached **(B)** HT-29 cells. Unexposed HT-29 **(C)** cells were used for additional control. **(A, B)** showed nearly identical uptake of the chemotherapeutic agent doxorubicin. There was therefore no difference in doxorubicin uptake between cells exposed to doxorubicin while in an adherent monolayer versus cells in a floating state in the eppendorf tube. Significance levels were indicated in the figure accordingly with: # (not significant (ns) black) no significant difference in the doxorubicin uptake or cell distribution and # (ns, red) no significant difference in the cell distribution. P values are indicated with *P < 0.05 and ^#^P >0.05.

All cells treated with doxorubicin showed nearly complete uptake of the chemotherapeutic agent. There was no significant difference in doxorubicin uptake between cells exposed to doxorubicin in an adherent monolayer versus cells in a floating state (**Figure 5A** versus **Figure 5B**). Both distribution patterns in the flow-cytometry for these two different exposure groups were nearly identical. Demonstrating that the there was no difference for uptake for cells while attached or floating. This experiment was performed in order to exclude differences in chemotherapy uptake as the potential cause for the different viability data in the previous experiments for these two groups. There were no doxorubicin-positive cells in the negative control group. A control group not exposed to doxorubicin was also tested as a further control demonstrating same cellular pattern however missing the doxorubicin signal (**Figure 5A** versus **Figures 5C, B** versus **Figure 5C**).

## 4 Discussion

Our findings indicate that chemotherapy—particularly oxaliplatin—is highly effective in disrupting and inhibiting cancer cell adhesion to surfaces, thereby markedly reducing the potential for new metastatic nodule formation. It is plausible that pre-adhesion chemotherapy exposure interferes with the synthesis of functional adhesive extracellular matrix (ECM) proteins, which are critical for cell surface attachment and sustained growth. Given the structural complexity of extracellular adhesive proteins, they may be especially susceptible to misalignment during transcription or to aberrant primary and secondary folding (13–15).

As previously noted, interaction with the ECM stabilizes cancer cells and promotes further proliferation (5–7). Although overall growth is suppressed by chemotherapy exposure, our data indicate that treatment during the pre-adhesion stage has a substantially greater inhibitory effect than treatment at later stages. Notably, the results suggest no significant difference in efficacy between the various time points of OX exposure prior to cell transfer. Chemotherapeutic exposure either during the attached stage or immediately after detachment results in reduced viability following transfer to the final well. Remarkably, intracellular uptake of the chemotherapeutic agent does not differ significantly between these two stages. The floating stage neither enhances nor diminishes drug influx into the cells. Nevertheless, overall inhibition of adhesion—leading to delayed growth and reduced viability—persists.

The addition of EDTA further inhibits cancer cell attachment to surfaces, thereby reducing overall cell viability to an even greater extent. Although data on the interaction of EDTA with the protein matrix are limited, available evidence suggests that EDTA can interfere with the proper folding of proteins (16). Clinically, overall inhibition of cancer cell adhesion could be exploited at various stages of the disease. For example, CRS for PM is routinely followed by HIPEC. The observed beneficial effects of HIPEC may, in part, be attributable to disruption of cancer cell adhesion following surgical removal of microscopic tumor nodules. Remaining floating cancer cells could thereby be significantly impaired in their ability to adhere to peritoneal surfaces. This interpretation is consistent with our IPM findings, which demonstrated a reduced capacity for metastatic colony formation.

Furthermore, empirical evidence indicates that early postoperative chemotherapy following surgical tumor resection is associated with improved outcomes in terms of cancer progression and, to some extent, survival rates (17, 18). This benefit may be attributable to the pronounced inhibition of adhesion in mobilized cancer cells resulting from iatrogenic mechanical detachment of individual cells or clusters from the primary tumor. These processes occur at the microscopic level, and their direct clinical manifestations typically become apparent only at later stages. The progression from micro metastases to radiologically detectable macroscopic nodules requires considerable time, as metastases generally need to reach a diameter of approximately 10 mm to be visible on computed tomography (CT) scans (19). Nevertheless, indirect systemic effects may be observed much earlier.

This represents an important phenomenon occurring on the peritoneal surface, where the widespread dissemination of cancer nodules may not yet be clearly detectable by conventional imaging modalities such as ultrasound or CT, despite a potentially high total tumor burden. The inhibition of cancer cell adhesion within the peritoneal cavity warrants further investigation to limit PM progression through ongoing metastatic spread. This multifocal dissemination is a major driver of increasing tumor burden in PM and significantly contributes to the deterioration of patients’ clinical status. Future research should also evaluate the potential role of agents outside the traditional chemotherapeutic drugs. Such agents could specifically target cell adhesion mechanisms and disrupt the adhesion process without exerting cytotoxic effects, potentially offering improved tolerability compared to conventional chemotherapy. The current study should encourage the search for a new class of drugs that specifically target cancer cell adhesion.

The limitations of this study are related to the used model. There is very little data and supporting studies looking at this particular problem. The well surface does not exactly resemble the circumstances within the body were a more complex interaction appears between cancer cells and the biological surface onto which the cancer cells attempt to adhere (20). The provide data are limited on HT-29 cells. There needs to be further studies if this effect is also present to the other entities and for other surfaces. There might be significant differences between cancer entities and location of adhesions. This could be further studied. The study does not further elaborate and examine the underlying mechanism that lead to the observed changes. This will need to be further explored in the future.

## 5 Conclusion

Exposure of HT-29 colorectal cancer cells to oxaliplatin during the floating stage markedly inhibits subsequent adhesion and growth, with a much greater effect than exposure during the later attached stage. This phenomenon is unlikely to be attributable to increased intracellular uptake of the chemotherapeutic agent. Surface adhesion inhibition can be further enhanced by EDTA and potentially other agents. Further investigations are warranted to optimize this approach for the suppression of PM progression, which remains a major clinical challenge in surgical oncology.

## Data Availability

The raw data supporting the conclusions of this article will be made available by the authors, without undue reservation.
